# The epidemiology and clinical correlates of HIV-1 co-receptor tropism in non-subtype B infections from India, Uganda and South Africa

**DOI:** 10.1186/1758-2652-15-2

**Published:** 2012-01-26

**Authors:** Quazi Ataher, Simon Portsmouth, Laura A Napolitano, Sybil Eng, Anna Greenacre, Andrew Kambugu, Robin Wood, Sharlaa Badal-Faesen, Randy Tressler

**Affiliations:** 1 Pfizer Inc., 235 East 42nd St, New York, NY 10017, USA; 2 Monogram Biosciences, 345 Oyster Point Boulevard, South San Francisco, CA 94080, USA; 3 Adult Infectious Diseases Clinic, Infectious Diseases Institute, Makerere University College of Health Sciences, PO Box 22418, Kampala, Uganda; 4 The Desmond Tutu HIV Research Centre, Faculty of Health Sciences, University of Cape Town, Anzio Road, Observatory, Cape Town, South Africa; 5 Clinical HIV Research Unit, Themba Lethu Clinic, Helen Joseph Hospital, Westdene 2092, Johannesburg, South Africa

**Keywords:** co-receptor, tropism, HIV-1 subtype, HIV-1 clade, CCR5 (R5), CXCR4 (X4), cellular factors

## Abstract

**Background:**

The introduction of C-C chemokine receptor type-5 (CCR5) antagonists as antiretroviral therapy has led to the need to study HIV co-receptor tropism in different HIV-1 subtypes and geographical locations. This study was undertaken to evaluate HIV-1 co-receptor tropism in the developing world where non-B subtypes predominate, in order to assess the therapeutic and prophylactic potential of CCR5 antagonists in these regions.

**Methods:**

HIV-1-infected patients were recruited into this prospective, cross-sectional, epidemiologic study from HIV clinics in South Africa, Uganda and India. Patients were infected with subtypes C (South Africa, India) or A or D (Uganda). HIV-1 subtype and co-receptor tropism were determined and analyzed with disease characteristics, including viral load and CD4^+ ^and CD8^+ ^T cell counts.

**Results:**

CCR5-tropic (R5) HIV-1 was detected in 96% of treatment-naïve (TN) and treatment-experienced (TE) patients in India, 71% of TE South African patients, and 86% (subtype A/A1) and 71% (subtype D) of TN and TE Ugandan patients. Dual/mixed-tropic HIV-1 was found in 4% of Indian, 25% of South African and 13% (subtype A/A1) and 29% (subtype D) of Ugandan patients. Prior antiretroviral treatment was associated with decreased R5 tropism; however, this decrease was less in subtype C from India (TE: 94%, TN: 97%) than in subtypes A (TE: 59%; TN: 91%) and D (TE: 30%; TN: 79%). R5 virus infection in all three subtypes correlated with higher CD4^+ ^count.

**Conclusions:**

R5 HIV-1 was predominant in TN individuals with HIV-1 subtypes C, A, and D and TE individuals with subtypes C and A. Higher CD4^+ ^count correlated with R5 prevalence, while treatment experience was associated with increased non-R5 infection in all subtypes.

## Background

Human immunodeficiency virus type-1 (HIV-1) is characterized by extensive genetic heterogeneity. Molecular epidemiologic studies have demonstrated that globally, the most prevalent forms of HIV-1 are subtypes (clades) C, B and A [1-3]. Subtype C, which accounts for almost 50% of all HIV-1 infections globally, predominates in sub-Saharan Africa and India [1-3]. Subtype B is the main genetic form in the Americas, Australia and western Europe; subtype A predominates in areas of central and eastern Africa (Kenya, Uganda, Tanzania and Rwanda) and in eastern Europe [1-3]; and subtype D is distributed mainly in east Africa, including Uganda [1]. HIV-1 subtypes differ by as much as 20-25% at the genetic level [2], and have varying biological characteristics, including differences in disease progression, pathogenicity, transmissibility and co-receptor usage [1,2,4-7].

Studies of HIV-1 co-receptor tropism, which have been conducted primarily in populations where subtype B infections predominate, have demonstrated a relationship between HIV-1 co-receptor use and disease stage. In general, early stages of infection and disease are characterized by greater prevalence of only C-C chemokine type 5 (CCR5)-tropic (R5) HIV-1, which has been associated with slower progression to AIDS [8-12]. The emergence of C-X-C chemokine receptor type 4 (CXCR4)-using virus (X4) has been associated with greater treatment experience and higher risk of death, and coincides with more rapid CD4^+ ^T-cell depletion and disease progression [6,8,9,12,13]. Some variants of HIV-1 can use either co-receptor (dual/mixed-tropic [DM] HIV-1); these can be found in all stages of infection, but are more common in infections of longer duration, with lower CD4^+ ^cell counts and higher viral loads [12-14]. Despite the emergence of X4-using variants in some patients, only R5 infection typically persists in the majority of patients. Nearly 50% of patients who die of HIV-1 disease have only R5 HIV-1 detectable at the time of their death, indicating that CCR5 remains a critical co-receptor throughout the course of HIV infection [12,15].

Although HIV-1 co-receptor usage and its relationship to disease stage have been studied in the developed world, where subtype B predominates, such relationships are less well understood for subtypes A, C and D. The R5 phenotype is predominant in subtype C HIV-1 infections, whereas X4-using virus has been reported infrequently, even in advanced disease. R5-using virus is more common in subtype A than subtype D HIV-1 infections, and a high proportion of subtype D infections shows D/M tropism throughout the course of disease [16-24]. However, some of these previous studies have been limited by small sample sizes.

The introduction of the CCR5 antagonist, maraviroc, for HIV-1 therapy [25] has increased interest in the epidemiology of tropism and relationships with HIV-1 subtype. A greater understanding of the tropism of non-B subtype HIV-1 is key for the optimal use of CCR5 antagonists in the treatment of these infections in the developing world, and HIV-1 prevention strategies, such as topical microbicides and systemic pre- or post-exposure prophylaxis. In addition, this information will be important for management of clinic populations in the developed world that include individuals with non-B subtype infections who have migrated from endemic countries [26]. HIV-1 tropism can be determined by genotypic and phenotypic methods. While genotypic assays may have lower specificity and sensitivity, retrospective analyses have found that they are comparable to phenotypic tropism assays for prediction of response to treatment with CCR5 antagonists, in populations pre-screened with a phenotypic assay [27,28].

The clinical development programme for maraviroc, the first-in-class CCR5 antagonist, used the Trofile^® ^phenotypic assay (Monogram Biosciences, South San Francisco, California) [29-31], which determines tropism via the expression of full-length *env *genes of multiple viruses isolated from patient plasma and can detect 10% of X4 variants with 100% sensitivity. More recently, a Trofile^® ^assay with enhanced sensitivity to improve detection of low-level X4-using variants has been developed that can detect 0.3% of these variants with 100% sensitivity. Otherwise, assay validation performance characteristics are equivalent between the original and enhanced Trofile^® ^assays [31]. The enhanced Trofile^® ^assay has been validated in a number of studies by re-testing the co-receptor tropism of clinical samples that were initially determined using the original assay [31-34]: in a re-analysis of samples from the Phase 3 MERIT study, which evaluated the efficacy of maraviroc in treatment-naïve patients with CCR5-tropic virus, 15% of enrolled patients (n = 106/721) were reclassified as having D/M virus with the enhanced assay [34].

The aim of this study was to estimate the prevalence of R5-, D/M-, and X4-tropic HIV-1 among isolates obtained from patients with HIV-1 subtype C infection from India and South Africa, and with subtype A/A1 and D infection from Uganda, and to explore the demographic and clinical characteristics associated with R5 infection. In addition, the study examined the ability of the Trofile^® ^assay to determine tropism of non-B subtypes of HIV-1, which previously had not been explored in a large study.

## Methods

### Study design

HIV-1-infected, antiretroviral therapy (ART) treatment-naïve (TN) and treatment-experienced (TE) viremic patients were recruited into this prospective, cross-sectional, epidemiologic study from HIV clinics in South Africa (four sites), Uganda (one site), and India (seven sites) in 2007 and 2008. Sites were selected if they had considerable experience with both HIV management and HIV research. The study protocol was approved by the institutional review board at each site.

Both TN and TE adults (aged 18 years or older) were eligible for enrolment in India and Uganda. In South Africa, where the MERIT study had previously been conducted in TN patients [35], only TE adults were eligible for inclusion in the present study. No patients were recruited from any other study.

Patients who had received less than 10 days of ART were considered to be TN; those who had experienced failure of at least one three-drug ART regimen were considered to be TE. To maximize the external validity and generalizability of the study, only one member of a known infection cluster (i.e., only one member of a family affected by HIV) was eligible for enrolment.

### Study procedure

At a single visit, the study was explained to patients, and verbal and written informed consent were obtained prior to conduct of any study procedures. Demographic, and HIV clinical history and treatment data were collected; blood was drawn and analyzed for CD4^+ ^and CD8^+ ^T cells using BD FACS™ CAP (Becton Dickinson and Company, Franklin Lakes, New Jersey), and for HIV-1 RNA levels using Amplicor HIV-1 Monitor™ UltraSensitive Assay (Roche Diagnostics, Indianapolis, Indiana).

For individuals with viral loads exceeding 500 copies/mL, HIV-1 subtype was determined based on reverse transcriptase and protease gene sequence (Monogram Biosciences). *Pol *subtyping was performed by generating three distinct sets of partial-length nucleotide sequences from patient-derived reverse transcriptase and protease genes. Genetic sequence comparison was determined by the Basic Local Alignment Search Tool algorithm, and subtype was resolved by a customized software package that was validated against the publically available tool on the National Center for Biotechnology Information web site at the National Institutes of Health. Samples determined to be HIV-1 subtypes of interest (A/A1 and D in Uganda; C in South Africa and India) were further tested for viral tropism.

HIV-1 co-receptor tropism was determined using the original Trofile^® ^assay [29,30] for samples collected from patients in South Africa and Uganda. The Indian cohort was enrolled after the South African and Ugandan cohorts, thus Indian patients were tested using the newly available enhanced Trofile^® ^assay, which had replaced the original assay. Samples were prepared for both the original and enhanced assays in the same way: 1 mL plasma samples underwent centrifugation and viral RNA was isolated, purified and subjected to polymerase chain reaction (PCR) amplification of the entire HIV envelope gene (*env*). Co-transfection of HIV *env *expression vectors and HIV-1 genomic vectors produced pseudoviruses containing full length *env *genes derived from patient virus populations. Tropism was determined by measuring the ability of the pseudovirus population to efficiently infect target cells co-expressing CD4 with either the CXCR4 or CCR5 co-receptor [29,31].

For commercial Trofile^® ^testing, up to 3 mL of plasma and up to three attempts at RNA PCR amplification are performed for each patient specimen. For this study of non-subtype B specimens, however, only a single attempt at amplification was made with 1 mL samples and primers that were optimized on subtype B specimens (hereafter referred to as "standard primers"). In the event that samples yielded a non-reportable tropism result, re-testing was performed using modified primers that were optimized for subtypes A, C and D but retained high performance for subtype B (hereafter referred to as "optimized primers"). All re-testing with optimized primers was performed using the same version of Trofile^® ^that was used for the initial 1 mL test. Thus for South African and Ugandan specimens, all re-testing was performed with the original assay, and for Indian specimens, all re-testing was performed with the enhanced assay.

### Sample size and statistical analysis

The study goal was to include at least 171 patients with reportable tropism results in each country, subtype and treatment experience stratum to provide approximately 5% precision for estimating prevalence of R5 in TN patients (anticipated to be 85%) and 7.5% precision for estimating prevalence of R5 in TE patients (anticipated to be 50%).

Demographic and HIV disease characteristics were summarized by country, subtype and treatment experience strata. The prevalence of R5, X4, and D/M HIV-1 among patients with reportable tropism results was estimated in each stratum. The relationship between R5 infection and the following variables was examined in univariate logistic regression analyses: age (per year); gender; CD4^+ ^cell count (per 50 cells/mm^3^); HIV-1 RNA level (per 10,000 copies/mL); ART experience status; time since HIV diagnosis (months); mode of transmission; and Centers for Disease Control and Prevention (CDC) HIV disease category. CD4^+ ^count and HIV-1 RNA level were also analyzed using quartiles. Variables significantly associated with tropism in univariate analyses (p < 0.05) were entered into multivariate logistic regression analyses, in which two-way interactions between all covariates were examined categorically. Backward elimination from a saturated model was used to determine the final predictive model.

## Results

### Patients

The demographic and clinical characteristics of patients with reportable HIV-1 tropism results are summarized in Table 1. All but four patients in South Africa and Uganda identified themselves as African: three self-identified as mixed race, and one as Caucasian. All but one patient in India (who self-identified as Asian) identified themselves as Indian. In all countries, nearly all patients reported heterosexual contact as the mode of HIV transmission.

**Table 1:** Demographics and clinical characteristics of patients with reportable tropism results

	India		South Africa	Uganda			
	
	Subtype C (N = 240)		Subtype C (N = 205)	Subtype A/A1 (N = 236)		Subtype D (N = 236)	
	
	TN (n = 174)	TE (n = 66)	TE (n = 205)	TN (n = 204)	TE (n = 32)	TN (n = 113)	TE (n = 23)
**Age: **mean (SD)	36 (8.2)	39 (7.5)	37 (7.9)	38 (8.7)	38 (8.7)	35 (8.4)	33 (8.3)

**Gender: **% male	65%	82%	41%	32%	31%	35%	22%

**Time since HIV diagnosis **(months): mean (SD)	23 (36.2)	68.4 (43.7)	48.9 (31.5)	37.7 (35.4)	62.8 (48.1)	32.6 (26.6)	46.6 (19.0)

**Number of treatment failures: **mean (SD)	NA	1.5 (1.6)	1.3 (0.58)*	NA	1.2 (0.4)	NA	1.0 (0.2)

**Absolute CD4^+ ^cell count (cells/μL): **mean (SD)	155.7 (128.5)	114.0 (110.1)	204.0 (146.1)*	334.3 (178.9)	83.4 (64.8)	361.0 (198.0)	109.3 (124.2)

**CD4^+ ^count: **range	3-775	6-504	1-681	5-1171	8-223	10-1255	1-589

**Viral load (log_10 _copies/mL): **mean (SD)	5.52 (5.46)	5.40 (5.42)	5.09 (5.55)	5.27 (5.33)	5.60 (5.20)	5.11 (5.19)	5.15 (5.22)

**Log viral load: r**ange	3.16-5.88	3.16-5.88	3.04-6.63	3.40-5.88	3.13-5.86	3.33-5.85	3.07-5.88

NA, not available; SD, standard deviation; TE, treatment-experienced; TN, treatment-naïve.* Missing 1; n = 204.

Haemophilia/coagulation disorder and/or blood transmission were reported as transmission factors by three patients (1.5%) in South Africa and two patients (1.1%) in India. Four patients (one in South Africa, one in Uganda, and two in India) reported other unspecified risk behaviours. Risk was not reported or identified by 21 patients (8.8%) in India, two patients (1.0%) in South Africa, and one patient (0.9%) in Uganda. Most patients in South Africa and Uganda were female; most Indian patients were male.

**Figure 1:**
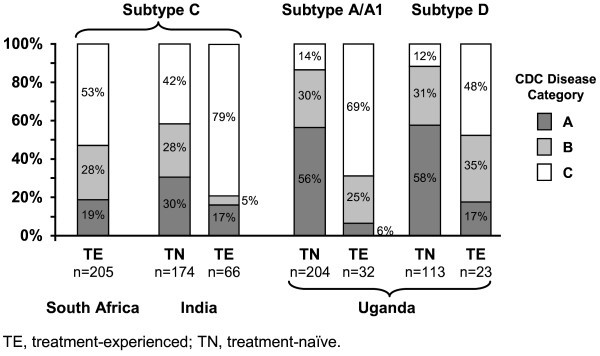
**Distribution of Centers for Disease Control and Prevention (CDC) human immunodeficiency virus disease category by treatment experience, subtype, and country**.

Indian patients had lower mean CD4^+ ^counts than South African and Ugandan patients; however, mean viral loads were consistent across the three countries (Table 1). TE patients were more likely (53-79%) to have CDC category C AIDS-defining events than TN patients (12-42%), regardless of country (Figure 1). Across treatment experience, the proportions of patients with CDC category C HIV disease were higher in India (79% of TE and 42% of TN patients) than in South Africa or Uganda (Figure 1).

### Subtype analysis

Samples for subtype analysis were collected from 307 patients (95 TE, 212 TN) in India, 678 (96 TE, 582 TN) in Uganda, and 297 (all TE) in South Africa. All samples with HIV-1 RNA exceeding 500 copies/mL were submitted for subtype testing. HIV-1 subtype could not be determined in 2.9%, 2.4%, and 5.9% of samples from India, Uganda, and South Africa, respectively, due to samples compromised by collection or handling problems and/or atypical genetic sequences (inability to generate HIV-1 pseudoviruses due to a rare genetic sequence within the patient's virus that is cleaved by the restriction enzyme used in the assay). Recruitment of TE patients with reportable tropism in Uganda and India, where HIV treatment is limited, was a challenge and required reducing the numbers of patients in these strata. TN samples were submitted for subtyping up until the enrolment goal of at least 171 patients had been reached.

Subtype C was predominant in India (overall, 280 [91%]: 202 [95%] TN and 78 [82%] TE patients) and South Africa (275 [93%] TE patients). The predominant subtypes in Uganda were A/A1 (overall, 324 [48%]: 285 [49%] TN and 39 [41%] TE patients) and D (overall, 203 [30%]: 167 [29%] TN and 36 [38%] TE patients). Other subtypes representing ≥ 1% of the Ugandan cohort were A/D (8%), AE (2.4%), C (2.2%), and Complex (1.5%).

### Tropism

**Figure 2:**
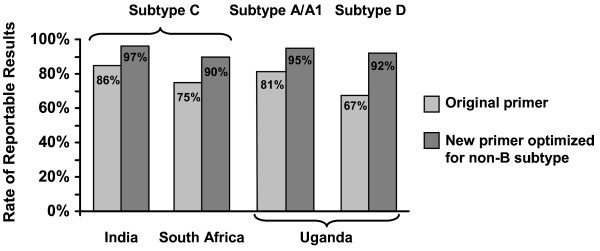
**Rates of reportable HIV-1 tropism results**.

Samples from patients with subtype C virus from India and South Africa and subtypes A/A1 and D from Uganda that were initially tested for co-receptor tropism using a single 1 mL aliquot of patient plasma yielded the following reportable results, respectively, when RNA amplification was performed with the standard primers: 240 (86%), 205 (75%), 236 (81%) and 136 (67%) (Figure 2). After re-testing all South African, Ugandan and Indian non-reportable samples using optimized primers for RNA amplification, the reportable rate improved to 90-97% in the different strata.

**Figure 3:**
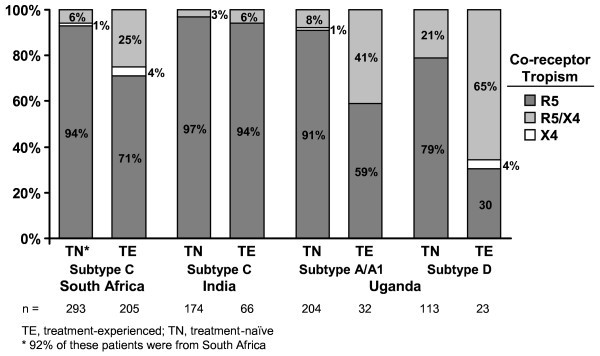
**Tropism distribution by treatment experience, country and subtype**.

Overall, most patients with reportable tropism results had only R5 HIV-1, but the proportion with R5 virus varied substantially by country, subtype and treatment experience (Figure 3). The proportion with only R5 virus ranged from 30% among TE Ugandan patients infected with subtype D to 97% among TN Indian patients infected with subtype C. D/M-tropic HIV-1 was less frequent, ranging from 3% among TN subtype C-infected patients in India to 65% among TE patients from Uganda with subtype D infection. X4 HIV-1 was rare and found in only eight TE patients from South Africa, and in two TN subtype A-infected patients and one TE subtype D-infected patient from Uganda.

Although co-receptor tropism varied according to country in TE patients with subtype C infection, R5 tropism was consistently high in TN patients with subtype C. R5 tropism was identified in 97% of TN patients with subtype C from India (Figure 3) and, in the MERIT study of maraviroc [35], a similar analysis identified R5 tropism in 94% of 293 TN patients with subtype C, of whom a large majority (92%) were South African.

**Figure 4:**
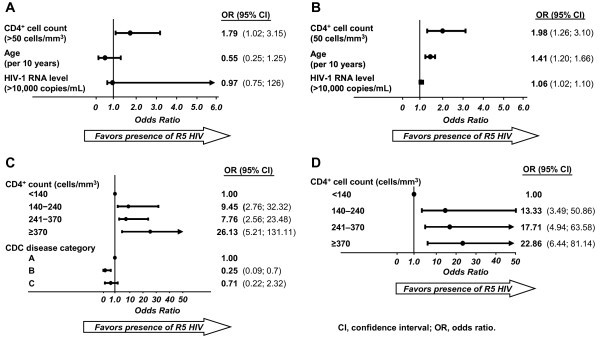
**Multivariate model for covariates significantly associated with R5 virus**. A. India subtype C (N = 238). B. South Africa subtype C (N = 205). C. Uganda subtype A/A1 (N = 236). D. Uganda subtype D (N = 136).

Variables significantly associated with tropism appear in Figure 4. Higher CD4^+ ^count was associated with an increased likelihood of R5 tropism in patients from both India and South Africa (Figures 4A and 4B). Additionally, in South African patients, older age and higher HIV-1 viral load predicted R5 tropism (Figure 4B). Among Ugandan patients infected with subtype A/A1 HIV-1, higher CD4^+ ^counts were associated with R5 tropism and CDC category B disease was negatively associated with R5 tropism (Figure 4C), whereas among patients infected with subtype D, only higher CD4^+ ^counts predicted R5 tropism (Figure 4D).

## Discussion

In this study, CCR5 was the predominant co-receptor used in non-B subtype HIV-1 in ART-naïve individuals from India and Uganda, and in TE individuals from South Africa. In contrast to prior reports, this study evaluated a large number (n = 817) of both TN and TE patients infected with non-B subtypes (C, A, D). There were difficulties recruiting TE patients in India and Uganda, which may indirectly reflect the availability of ART, estimated by the World Health Organization to reach only 31% of HIV-infected persons in these countries [36].

Most (83%) study subjects with reportable tropism results had only R5 HIV-1 detectable, including 90% of TN and 72% of TE patients. However, tropism distribution varied by subtype, CD4^+ ^cell count and treatment experience, which can be considered a marker of advanced disease. Indian subjects with subtype C virus were almost exclusively infected with R5 regardless of their CD4^+ ^count, HIV-1 disease status, or treatment experience. The prevalence of R5 virus was similarly high in TN South African patients with subtype C infection but, while most common and relatively well-preserved, was somewhat lower in the South African TE cohort of this study (71%). The difference observed between R5 prevalence in India and South Africa may be a result of differences in CD4+ cell count. Compared with the Indian cohort, there was a higher proportion of individuals in the South African cohort with a D/M phenotype (25%) despite similar time since HIV diagnosis and similar exposure to prior therapies. Exclusive use of X4 was rare (4%).

Our findings are consistent with previous reports of subtype C strains from India and elsewhere, where R5 HIV-1 is maintained even in advanced stages of disease. For example, some similarities can be observed between the findings of the present study and a previous study that assessed co-receptor tropism of HIV-1 strains in China: mainly that R5 tropism was seen exclusively in subtype C infections, and remained stable over time [24]. These data highlight the persistence of CCR5 tropism even in long-term infections, possibly related to differences within the *env *variable loops [37], and contrasts with other subtypes, especially subtype B, where X4 HIV-1 is more frequent during later stages of infection [6,8-10,16,18,21,37-40].

The distribution of HIV-1 subtypes in India and South Africa was consistent with published epidemiologic data [6]. However, this study found more subtype diversity within the Ugandan cohort than previously reported [41,42]. Despite the existence of multiple subtype variations in Uganda, the predominant subtypes were A/A1 (49.0%) and D (28.7%), and there were no marked differences in demographics or HIV disease characteristics. Among Ugandan subjects with subtype A/A1 virus, R5 infection predominated in TN individuals but was reduced in TE individuals, similar to the pattern seen with subtype B. While subtype D TN patients were predominantly R5, X4-using virus (D/M and X4) was detected in nearly 70% of the small number of TE patients. This agrees with earlier reports, where Ugandan individuals infected with subtype D virus showed predominantly D/M tropism [6,16-19,21,22].

Multivariate analyses of other cohorts have identified higher CD4^+ ^cell count as a predictor of R5 infection in both TN and TE patients with subtype B and C HIV-1 infections [43,44]. In the present study, multivariate analysis identified CD4^+ ^count as a significant predictor of CCR5 tropism for subtypes C, A/A1, and D. In Ugandan patients with subtype A/A1, R5 use was negatively associated with the presence of CDC category B disease, with a negative trend for category C.

This study provided an opportunity to assess the ability of the Trofile^® ^assay to provide results in different non-B subtype HIV-1 viral subtypes collected in developing countries. While initial testing using standard amplification primers with just a single 1 mL aliquot of patient plasma yielded a lower reportable rate (67-86%) than that expected from experience with subtype B virus, non-reportable samples that were re-tested with the original Trofile^® ^assay (South Africa and Uganda) or enhanced assay (India) using modified primers optimized for non-B subtype virus, yielded a much higher percentage of reportable results (90-97%), and the distribution of R5 and non-R5 HIV-1 did not change. These improvements in amplification primers - which also retain high performance for subtype B specimens and are currently the primers used regularly for RNA amplification in the Trofile assay - may be advantageous when Trofile testing is used in clinical populations where non-subtype B virus is common [45].

Globally, these data offer guidance on the potential use of CCR5 antagonists and tropism tests in resource-poor clinical settings. Since HIV transmission appears to be mostly associated with R5 HIV-1 regardless of subtype [46-48] and a large majority of chronic HIV-1 infections in India and South Africa are R5, CCR5 antagonists may have a role in prevention efforts: for example, use as microbicides or in pre- and post-exposure prophylaxis. Combining a CCR5 antagonist with another ARV agent active against all or X4-using viruses may improve the potential utility of CCR5 antagonists in ARV-based HIV-1 prevention strategies. In the context of evaluating patients for CCR5 antagonist therapy, the high proportions of R5 HIV-1 in both TN (97%) and TE (94%) patients in India suggests that alternative methods for determining co-receptor status could be explored in this setting. Tropism could potentially be tested using genotypic or phenotypic co-receptor tropism tests and/or virologic response to short-term CCR5 antagonist monotherapy.

## Conclusions

This study examined the correlates of HIV-1 co-receptor tropism in virus from individuals with non-B subtype HIV-1 infection from India, Uganda and South Africa. While only R5 virus was detectable in the majority of TN individuals infected with subtypes C, A and D, infection with only R5 virus was maintained, albeit at lower levels, in TE individuals. However, R5 prevalence varied by subtype: patients with subtype C, especially in India, were almost exclusively infected with R5, while subtype D patients were more frequently infected with D/M viruses. The high prevalence of R5 HIV-1 in subtype C infections makes a CCR5 antagonist an attractive option for ART-based prevention strategies in areas where subtype C predominates. While a higher CD4^+ ^cell count was highly correlated with CCR5 use regardless of subtype, a further understanding of HIV-1 co-receptor use in individuals and populations infected with non-subtype B HIV-1 is still needed. Given the heterogeneity of HIV-1 co-receptor use in non-subtype B HIV-1, there remains a need for prospective determination of HIV-1 tropism prior to the initiation of CCR5 antagonist therapy in geographic locations where the burden of infection is greatest.

## Competing interests

Quazi Ataher and Simon Portsmouth are employees of Pfizer Inc. and own Pfizer stock. Sybil Eng, Anna Greenacre and Randy Tressler were employees at Pfizer Inc. at the time that this study was conceived and conducted. Randy Tressler currently owns Pfizer stock and is eligible for a Pfizer pension. Laura Napolitano is an employee of Monogram Biosciences and its parent company, LabCorp, and owns LabCorp stock. Andrew Kambugu has received honoraria from Abbott. Robin Wood has received advisory fees from Merck, GlaxoSmithKline, Abbot and Tibotec. Sharlaa Badal-Faesen declares that she has no competing interests.

## Authors' contributions

QA conducted analyses of clinical samples and led manuscript preparation. LAN led and performed the standard and optimized primer data analysis. SP and SE participated in data interpretation. AG conducted statistical analyses of study data. AK, RW and SBF participated in study design, enrolled study subjects and collected clinical samples for analysis. RT conceived of the study, and participated in its design and in data interpretation. All authors reviewed and edited the manuscript.
